# Diagnostic accuracy of the vegetative and minimally conscious state: Clinical consensus versus standardized neurobehavioral assessment

**DOI:** 10.1186/1471-2377-9-35

**Published:** 2009-07-21

**Authors:** Caroline Schnakers, Audrey Vanhaudenhuyse, Joseph Giacino, Manfredi Ventura, Melanie Boly, Steve Majerus, Gustave Moonen, Steven Laureys

**Affiliations:** 1 Coma Science Group, Cyclotron Research Center, University of Liege, Belgium; 2 New Jersey Neuroscience Institute, Edison, NJ, USA; 3 CTR Neurorehabilitation Centre, Université Libre de Bruxelles, Brussels, Belgium; 4 Department of Neurology, Centre Hospitalier Universitaire Sart Tilman, University of Liege, Belgium; 5 Department of Cognitive Sciences, Experimental Psychology and Cognitive Neuroscience Research Unit-URPENC, University of Liege, Belgium

## Abstract

**Background:**

Previously published studies have reported that up to 43% of patients with disorders of consciousness are erroneously assigned a diagnosis of vegetative state (VS). However, no recent studies have investigated the accuracy of this grave clinical diagnosis. In this study, we compared consensus-based diagnoses of VS and MCS to those based on a well-established standardized neurobehavioral rating scale, the JFK Coma Recovery Scale-Revised (CRS-R).

**Methods:**

We prospectively followed 103 patients (55 ± 19 years) with mixed etiologies and compared the clinical consensus diagnosis provided by the physician on the basis of the medical staff's daily observations to diagnoses derived from CRS-R assessments performed by research staff. All patients were assigned a diagnosis of 'VS', 'MCS' or 'uncertain diagnosis.'

**Results:**

Of the 44 patients diagnosed with VS based on the clinical consensus of the medical team, 18 (41%) were found to be in MCS following standardized assessment with the CRS-R. In the 41 patients with a consensus diagnosis of MCS, 4 (10%) had emerged from MCS, according to the CRS-R. We also found that the majority of patients assigned an uncertain diagnosis by clinical consensus (89%) were in MCS based on CRS-R findings.

**Conclusion:**

Despite the importance of diagnostic accuracy, the rate of misdiagnosis of VS has not substantially changed in the past 15 years. Standardized neurobehavioral assessment is a more sensitive means of establishing differential diagnosis in patients with disorders of consciousness when compared to diagnoses determined by clinical consensus.

## Background

Differentiating the vegetative (VS) from minimally conscious state (MCS) is often one of the most challenging tasks facing clinicians involved in the care of patients with disorders of consciousness (DOC). Whereas VS is characterized by the return of arousal without signs of awareness [1], MCS is defined by the presence of inconsistent but reproducible goal-directed behaviors (e.g. response to command, verbalizations, visual pursuit, etc.) [2]. Behavioral assessment remains the "gold standard" for detecting signs of consciousness and, hence, for determining diagnosis [3]. However, behavioral assessment is complicated by the presence of motor impairment, tracheotomy, fluctuating arousal level or ambiguous and rapidly habituating responses [4]. Previous studies have shown that 37 to 43% of patients diagnosed with VS demonstrated signs of awareness [5,6]. Misdiagnosis can lead to grave consequences, especially in end-of-life decision-making [7]. Contrary to patients in VS, those in MCS retain some capacity for cognitive processing and activate similar brain networks relatives to controls following painful stimulation; suggesting that they can experience pain [8,9]. Moreover, the prognosis of patients in MCS is significantly more favorable relative to those in VS [10]. End-of-life decisions, therefore, are likely to be influenced by whether one is diagnosed with MCS or VS. In 2002, criteria were proposed to characterize MCS and identify behaviors that signal emergence from this state [2]. In view of the availability of the MCS criteria, the incidence of misdiagnosis of VS should be lower than the rates reported before these criteria were established [11]. However, no recent studies have investigated the accuracy of this grave clinical diagnosis. Over the last 15 years, specialized neurobehavioral rating scales have been developed to provide a reliable and valid means of detecting signs of consciousness. There are significant differences among these scales, however, with respect to diagnostic sensitivity [3]. The Coma Recovery Scale-Revised (CRS-R) was developed specifically to differentiate MCS from VS [12]. We recently showed that the proportion of patients diagnosed with MCS by the CRS-R was significantly higher as compared to other neurobehavioral scales such as the Glasgow Coma Scale [13], the Full Outline of UnResponsiveness [14] and the Wessex Head Injury Matrix [15]. These results suggest that the type of assessment tool used is crucial to accurate diagnosis [16,17]. In this study, we compared consensus-based diagnoses of VS and MCS to those based on the CRS-R, a well-established standardized neurobehavioral rating scale.

## Methods

Participating centers were intensive care and neurology units as well as neurorehabilitation centers, part of the Belgian federal network for care of patients in VS and MCS. All the patients included in this study were recruited according to pre-arranged inclusion/exclusion criteria. Inclusion criteria were a) severe acquired brain injury causing disturbance in consciousness, b) no neuromuscular function blockers and no sedation within the prior 24 hours and c) periods of eye opening (indicating preserved sleep-wake cycles and emergence from coma). Exclusion criteria were a) documented history of prior brain injury, b) premorbid history of developmental, psychiatric or neurologic illness resulting in documented functional disabilities up to time of the injury and c) acute illness. Additionally, the following clinical information was collected from the medical file of each patient: age, gender, past medical history, medication, time since onset and etiology.

We used a standardized neurobehavioral assessment scale to determine patients' level of consciousness: the Coma Recovery Scale-Revised (CRS-R) [12]. The CRS-R assesses auditory, visual, verbal and motor functions as well as communication and arousal level. The total score ranges between 0 (worst) and 23 (best). The CRS-R has shown superior performance in detecting VS and MCS compared to other scales [12,16,17]. Post-comatose patients were assessed once with the CRS-R by experienced raters (CS or AV). Relying on the Aspen criteria [2], we operationalized the definitions of VS, MCS and emergence from MCS using the items on the scale that were designed for this purpose. CRS-R-derived diagnostic criteria are mentioned in Table 1. We compared the diagnosis derived from the CRS-R assessments performed by the research team (CS or AV) to the clinical consensus diagnosis. The clinical consensus diagnosis was based on daily behavioral observations and included observations made within the last 24 hours by a clinical team comprised of physicians, psychologists, speech therapists, occupational therapists, physiotherapists and nurses. The research team was not involved in the clinical consensus diagnoses. The physicians recorded the clinical consensus diagnosis according to the observations reported by each member of the clinical team during structured but also unstructured team meetings and, in all cases, communicated this diagnosis to the research team prior to conducting the CRS-R assessment. The research team was hence not masked to the clinical consensus diagnoses. When all the clinical staff agreed, diagnosis was deemed VS or MCS. When even one person disagreed, the diagnosis was deemed 'uncertain'. Patients thought to have emerged from MCS based on consensus diagnosis were not assessed on the CRS-R. All patients were assessed once by the research team and were assigned a diagnosis of VS, MCS or emerged from MCS. Differences in diagnosis relative to length of time post-injury (acute vs. chronic) and etiology (traumatic vs. non-traumatic) were assessed using Chi square test, thresholded for significance at p < 0.05. The study was approved by the Ethics Committee of the University of Liège. Informed consent was obtained from each patient's legal surrogate.

**Table 1:** CRS-R's diagnostic criteria for vegetative (VS) and minimally conscious (MCS) states and emergence from MCS

VS	Auditory ≤ 2 *AND *Visual ≤ 1 *AND *Motor ≤ 2 *AND *Oromotor/Verbal ≤ 2 *AND *Communication = 0 *AND *Arousal ≤ 2
MCS	Auditory = 3–4 *OR *Visual = 2–5 *OR *Motor = 3–5 *OR *Oromotor/Verbal = 3 *OR *Communication = 1
Emergence from MCS	Motor = 6 *OR *Communication = 2

## Results

The data were collected between October 2005 and January 2007. We enrolled 103 patients in this study (74 in acute care and neurology units and 29 in neurorehabilitation centers). There were 71 males (69%) and mean age was 55 ± 19 years. Forty-six patients (45%) were in the acute recovery period (mean 12 ± 7 days) and 57 were in the chronic stage (55%) (mean 22 ± 52 months) [16]. The following etiologies were included [9]: traumatic head injury (n = 39), postanoxic-ischemic encephalopathy (n = 31), ischemic or hemorrhagic stroke (n = 16), aneurysmal subarachnoid hemorrhage (n = 6), metabolic encephalopathy (n = 5) and miscellaneous acute neurological conditions (n = 6).

Of the 44 patients with a clinical consensus diagnosis of VS, the CRS-R detected signs of awareness in 18 patients (41%). Misdiagnosis was greater for chronic (14 out of 29; 48%) than for acute patients (4 out of 15; 27%) (Chi^2 ^= 7; p < .01; CI (95%) = 39.55 to 60.45). Behavioral signs of consciousness detected by the CRS-R in patients misdiagnosed by clinical consensus primarily included purposeful eye movements (visual fixation: n = 3; visual pursuit: n = 5). In patients assigned a clinical consensus diagnosis of MCS (n = 41), 10% (n = 4) met criteria for emergence from MCS. All four of the patients found to have emerged from MCS were in the chronic stage. Finally, among the 18 patients with an uncertain clinical consensus diagnosis, 16 manifested signs of consciousness (89%; 8 acute and 8 chronic) when examined with the CRS-R. The clinical signs most often encountered in the latter patients were again purposeful eye movements (visual fixation, n = 4; pursuit, n = 2) (see Table 2). Finally, we observed no significant difference in diagnostic accuracy when traumatic cases were compared with non-traumatic cases.

**Table 2:** Behavioral signs of consciousness found in patients misdiagnosed with VS and MCS or with uncertain clinical consensus diagnosis.

**Behavior**	**VS**	**MCS**	**Unsure of diagnosis**
1 – response to verbal order	4	*	4
2 – purposeful eye movements	8	*	6
3 – automatic motor response	1	*	1
4 – pain localization	1	*	1
5 – several criteria for MCS	4	*	4
6 – communication	*	1	*
7 – functional object use	*	1	*
8 – several criteria for EMCS	*	2	*

Total	18	4	16

Notice: EMCS = emergence from MCS; * Non-appropriate; several criteria for MCS = presence of several criteria such as response to verbal order, purposeful eye movements, automatic motor response and/or pain localization; several criteria for EMCS = presence of communication and functional object use.

## Discussion

In this geographically-diverse sample drawn from a Belgian federal network of brain injury treatment centers, the rate of misdiagnosis of VS (41%) is roughly equivalent to rates reported in the U.S. and U.K. before the criteria for MCS were published [5,6]. Misdiagnosis occurred most often as the result of failure to detect purposeful eye movements (i.e., visual fixation and pursuit), in line with previous studies [5]. Moreover, our study suggests that the majority of cases with an uncertain diagnosis are in MCS (89%), not in VS. Finally, a false negative diagnosis of MCS was noted in 10% of cases that had emerged from this condition.

One could argue that the false negative consensus-based MCS diagnoses actually represent false positive CRS-R diagnoses of MCS. This possibility cannot be excluded but is also not easily resolved. As false positive errors increase, specificity decreases. However, in the context of a weak gold standard, false positives may not actually reflect diagnostic errors. If we consider the clinical consensus diagnosis as the gold standard, then false positive errors on the comparison measure (i.e., diagnosis of MCS) will result in lower specificity. Such false positive errors may, however, be due to the superior capacity of the comparison measure to detect the behavior of interest. In this case, the CRS-R, a standardized measure, captured more behavioral signs of consciousness relative to the collective impression of the medical team. One could also argue that there was a bias in favor of the research team's diagnostic accuracy as the researchers were not blind to the consensus diagnosis. The research team's knowledge of the clinical consensus diagnosis may hence have overestimated the sensitivity of CRS-R to detect signs of consciousness. However, the CRS-R requires replication of behavioral responses before scoring them as present (e.g., a response to verbal order is considered scoreable if the appropriate behavior is observed on 3 out of 4 trials) and, as such, decreases the risk of a false positive diagnosis. Future studies including blind assessment could be performed in order to comfort our results.

Spontaneous recovery is unlikely to explain our results as the clinical consensus diagnosis included behaviors observed by the medical staff within the prior 24 hours and was provided just before the CRS-R assessment. It is more likely that the examiners' reliance on unstructured bedside observations contributed to the high rate of misdiagnosis of VS patients. Indeed, it has been suggested that misdiagnosis is influenced by the use of a standardized behavioral tool [4]. In this study, we compared the accuracy of diagnoses based on standardized behavioral assessment using the CRS-R with consensus-based diagnoses established by the medical team following qualitative observations. Unlike traditional bedside assessment, the CRS-R guards against misdiagnosis by incorporating items that directly reflect the existing diagnostic criteria for MCS, and by operationalizing scoring criteria for the identification of behaviors associated with consciousness. Standardized assessment approaches may hence mitigate the tendency to miss signs of consciousness that may arise when the diagnosis is based solely on routine bedside examination. In cases with ambiguous behavioral findings, the failure to employ a standardized behavioral tool may increase the likelihood of misdiagnosis. Reliance on qualitative (versus standardized) assessment could also explain the higher rate of misdiagnosis observed for VS and MCS patients in both chronic and acute care settings. There is evidence to support this premise. Data regarding the use of a standardized behavioural scale were collected for each centre involved in this study. The behavioural scales' scores were not reported to the research team but were taken in account for the clinical consensus diagnosis. All 46 patients in the acute setting were evaluated with the Glasgow Coma Scale, a standardized assessment tool, and only 4 of these cases (10%) were misdiagnosed. In contrast, of the 57 chronic patients, 30 were not assessed using a standardized measure and 9 cases (30%) were misdiagnosed.

Finally, for uncertain diagnoses, we did not collect information on who disagreed and how frequently. While it would be helpful to know if uncertain diagnoses were due to the ambiguity of patient's responses or to the observational skills of the examiner, the aim of this study was to assess the misdiagnosis rate rather than explain the causes of misdiagnosis.

## Conclusion

Despite the importance of diagnostic accuracy and advances in the past 15 years, the rate of misdiagnosis among patients with disorders of consciousness has not substantially changed. Although early detection of signs of consciousness is crucial not only for daily management (particularly, pain treatment), end-of-life decisions [7] and prognosis (i.e., patients in MCS have significantly more favorable outcomes as compared to those in VS [10]), clinicians should recognize that diagnoses established during the acute stage tend to be transitional and may change over time as the injury sequelae resolve. The results of this study suggest that the systematic use of a sensitive standardized neurobehavioral assessment scale may help decrease diagnostic error and limit diagnostic uncertainty. Future studies should investigate other factors influencing diagnostic accuracy.

## List of abbreviations used

The list of abbreviations used is the following: CRS-R: (Coma Recovery Scale-Revised); VS: (Vegetative State); MCS: (Minimally Conscious State).

## Competing interests

Dr Giacino participated in the development of the CRS-R and was involved in the review and discussion of the study results but did not participate in the study design, data collection or analyses.

## Authors' contributions

CS made substantial contributions to conception and design, acquisition of data, analysis and interpretation of data as well as in drafting the manuscript; AV was implied in collecting behavioural data, in the interpretation of data as well as in drafting the manuscript; JG gave critical revision of the manuscript for important intellectual content; MV, MB, SM and GM were involved in drafting the manuscript; SL made substantial contributions to design and supervised this study. All authors read and approved the final manuscript.

## Pre-publication history

The pre-publication history for this paper can be accessed here:


